# Allergy to beer – case report

**DOI:** 10.1186/2045-7022-5-S3-P62

**Published:** 2015-03-30

**Authors:** Natalia Ukleja-Sokolowska, Ewa Gawronska-Ukleja, Magdalena Zbikowska-Gotz, Zbigniew Bartuzi

**Affiliations:** 1 Clinic of Allergy, Clinical Immunology and Internal Diseases, Collegium Medicum, UMK, Bydgoszcz, Poland

## Introduction

Beer is made mainly from barley, hops and yeast. Each of these ingredients may be the source of hypersensitivity.

## Material and methods

Patient, male, 50 years old, treated in the Outpatient Clinic of Allergic Diseases because of bronchial asthma. On a follow up visit patient was complaining of watery rhinorrhea, swelling and itching of hands and feet, generalized urticaria. Careful interview reveled that patient usually drinks a beer in the afternoon and symptoms worsen immediately after ingestion. During diagnosis patient had skin prick test (SPT) with inhalatory and food allergensusing a set from Allergopharma. We also determined the level of allergen specific IgE against Dermatophagides pteronissinus and Dermatophagoides farine, Alternata alternata, Cladosporium, Aspergillus, Hops and Barley. Patient had prick by prick test with three brands of beer, fresh and dried hops, baking yeast, barley and hopped liquid malt extract.

## Results

Patient had positive SPT for D. pteronissinus 8mm, D. farinae 5mm. Eleveted level of asIgE against Alternaria in II class (1.6 kU / ml). Prick by prick test with three brands of beer were positive (I - 7mm, II - 8 mm, III - 4mm). Prick test with hopped liquid malt extract was also positive - 6mm and native hops: dried - 7mm and fresh - 8mm. The level of asIgE against barley and hops was not elevated.

## Conclusion

The patient was diagnosed with allergy to beer ingredients and was recommended to exclude it from the diet. Patients condition improved, skin lesions completely disappeared. Attempts of beer consumption were followed with recurrence of symptoms within 30 minutes of ingestion.

## Consent

Written informed consent was obtained from the patient for publication of this abstract and any accompanying images. A copy of the written consent is available for review by the Editor of this journal.

